# Medical cannabis-related stigma: cancer survivors’ perspectives

**DOI:** 10.1007/s11764-022-01297-7

**Published:** 2022-11-26

**Authors:** Manan M. Nayak, Anna Revette, Peter R Chai, Kristina Lansang, Timothy Sannes, Stephanie Tung, Ilana M. Braun

**Affiliations:** 1 Department of Psychosocial Oncology and Palliative Care, Dana-Farber Cancer Institute, Boston, MA USA; 2 Phyllis F. Cantor Center for Research in Nursing and Patient Care Services, Dana-Farber Cancer Institute, 450 Brookline Avenue, Boston, MA LW-51902215 USA; 3 Survey and Qualitative Methods Core, Department of Population Sciences, Dana-Farber Cancer Institute, Boston, MA USA; 4 Department of Emergency Medicine, Brigham and Women’s Hospital, Boston, MA USA; 5 The Koch Institute for Integrated Cancer Research, Massachusetts Institute of Technology, Cambridge, MA USA; 6 The Fenway Institute, Boston, MA USA; 7 School of Nursing, University of California Los Angeles, Los Angeles, CA USA; 8 Harvard Medical School, Boston, MA USA

**Keywords:** Medical marijuana, Cannabis, Social status, Social stigma, Communication, Patient care team, Delivery of healthcare

## Abstract

**Background:**

Although the vast majority of medical cannabis laws in the USA includes cancer as a qualifying condition and medical cannabis-related stigma influences decision-making regarding the botanical, few studies have explored the phenomenon in oncology. Early findings indicated oncologic cannabis-related stigma to be quite widespread.

**Methods:**

Semi-structured interviews with 24 adults with cancer histories using medical cannabis were analyzed using the Health Stigma and Discrimination Framework.

**Results:**

Sixteen out of 24 participants discussed medical cannabis-related stigma in some depth. The phenomena emerged as more pervasive in medical than personal/professional domains and was internalized as well as experienced directly. It led some participants, but not others, to practice partial or complete secrecy.

**Discussion:**

Taken together, our findings suggest that, while medical cannabis-related stigma remains widespread and led some study participants to alter behavior, an early shift in ethos towards greater medical cannabis acceptance could be underway. If so, this transition may be occurring more rapidly in non-medical than in clinical settings.

**Conclusion:**

Cancer survivors may experience heightened medical cannabis-related stigma in the clinic as compared to their personal/professional lives. Healthcare providers who depend on patient transparency when gathering medical histories and devising care plans may wish to neutralize perceptions of medical cannabis-related stigma.

## Introduction

Medical cannabis is nonpharmaceutical, herbal cannabinoid products that individuals use for medical purposes, sometimes authorized by clinical professionals in compliance with state laws. Thirty-seven states in the United States (U.S.) permit medical cannabis use and a cancer diagnosis qualifies for medical cannabis in nearly every such state law. For this reason, understanding the role medical cannabis plays in oncology should be of import to all clinicians. Defined as a social process of stereotyping, labeling and grouping, stigma triggers loss of social status and discrimination, which in turn influences health and other outcomes [1, 2]. Although stigma associated with medical cannabis may influence decision-making regarding the botanical, only one stigma-related study (to our knowledge) has focused on an oncologic population. In it, stigma emerged as a significant barrier to medical cannabis use by cancer survivors [3].

Outside of oncology, the limited number of studies examining medical cannabis-related stigma have suggested that the phenomenon is pervasive, if gradually shifting [3–9]. Testimony from individuals using or considering use of medical cannabis indicated a perception that their communities view the practice as deviant and that stigma exists in an internalized form (e.g., assumed) in addition to an externalized (e.g., directly experienced) one [2]. Both forms seem to impact patient transparency about use with healthcare teams, as well as with social networks [1, 3, 4].Of note, a recent study (out of Canada) identified less stigma than earlier investigations. The paper’s authors attributed the finding to liberalization of Canadian medical cannabis laws, allowing medical cannabis use greater social acceptance and legitimacy [5, 10].

The studies above were conducted regionally (e.g., Canada/Israel/California) and differing cultural norms across geographies may have limited generalizability. Our study aimed for broader applicability by including a geographically diverse U.S. sample as well as individuals across the disease trajectory and aimed to understand the drivers and facilitators of oncologic cannabis stigma, guided by the Health Stigma and Discrimination Framework [11]. This multidimensional, theory- and evidence-based framework was selected to allow exploration of medical cannabis-related stigma at an individual as well as macro levels.

## Materials and methods

Complete methodology is described in the initial paper [12]. In brief, researchers selected eight geographically/culturally diverse states/districts with legal medical cannabis: Arizona, California, Florida, Illinois, Massachusetts, Oregon, New York, and the District of Columbia. Through state-sanctioned medical cannabis dispensaries, researchers recruited 24 individuals 21-years or older, certified to use medical cannabis and with physician-verified cancer histories. Participants received $75 honoraria for approximately 45-min interviews. Written informed consent enabled confidential, audio-recorded phone interviews which adhered to a semi-structured interview guide that included prespecified inquiries pertaining to stigma: *Have you experienced stigma around [medical cannabis]? If yes, from where/whom? How, if at all, has stigma affected your decisions around using [*medical cannabis*]?* Between April 2017 and March 2019, recruitment occurred in phases to ensure adequate capture of emergent themes. A qualitative research expert (AR) coded and analyzed transcripts using a multi-stage thematic analysis that combined prefigured and emergent codes and incorporated aspects of grounded theory, more applied framework analysis and a Health Stigma and Discrimination Framework [11, 13–16]. Each stage of coding/analysis was iteratively designed and discussed by an interdisciplinary research team (I. M. B., M. M. N., A. R.) to address trustworthiness in approach/interpretations and resolve conflicts. Recruitment ceased following achievement of thematic saturation. The Dana-Farber Cancer Institute Institutional Review Board approved this study (Protocol 15-449).

## Results

Of 24 participants, 16 (67%) were women and the median age was 57 years [range: 30–71 years]. Eleven (46%) resided in the Eastern U.S.; seven (29%) in the Western; and six (25%), in the Midwestern. Twelve (51%) had stage IV or metastatic disease, eight (33%) early-stage diagnoses, and four (17%) were in remission. Participant used medical cannabis for symptom management including for pain (*n* = 19), nausea/poor appetite (*n* = 14), anxiety/depression (*n* = 13), and poor sleep (*n* = 10), and more than half also used the botanical as treatment for cancer itself. Additional demographics are described elsewhere [12].

### Medical cannabis-related stigma more pervasive in the medical than personal/professional domain

Manifestations of stigma experiences and practices frequently emerged unprompted during interviews (Table 1). Participants noted the phenomenon in two arenas: the medical (which we will define as organized provision of healthcare to individuals or a community) and the personal/professional (which we will define as private life, relationships, career). While nearly all participants perceived some level of medical cannabis-related stigma in at least one of these domains, stigma in medical settings was considerably more pervasive.

**Table 1:** Exemplar quotations

Theoretical Framework	Theme	Subtheme	Exemplar Quotations
Stigma Manifestations: Experiences and Practices	Presence of stigma	Global	*It bothers me. It bothers me because there’s such a bad name attached to marijuana. So the minute you say to somebody, ‘Yeah, I take cannabis oil’: In their mind they’re going, ‘Oh my God…Why* [*are they*] *taking that stuff?’* *There’s a stigma out there, too where if you use cannabis, then you’re a pothead*.
		In personal/professional domain	[*My family*] *probably thinks … I’m a junkie.*^†^
		In medical domain	*Stigma? No. They’re the reasons my bones collapsed, because when I told him I was in pain, he said, oh. I’m a drug user. I use marijuana. I told them about that I had done this research. He’s a doctor. Right? Talk about help. Talk about your body. Talk about anything that’s real. Oh, yeah. He says, I can’t write that, they’re illegal.* *Because most doctors: You mention cannabis: They shut right up. They don’t say two words to you. They don’t give you an opinion: Nothing. They just shut right up.* *I didn’t really think about it… but then I went to a cancer conference, and I mentioned how much* [*MC*] *helped me, and people were… giggling or a little bit almost like immature about it.* *There’s an embarrassment that people have when they talk to you about medical marijuana. Even I find it consistently inconsistent throughout health care, which is that people — some people — like, not that they laugh, but they just — they have a kind of moral opposition to it. I’m reading into it. I’m not quite sure if that’s really the case.* ^†^
	Absence of stigma	In personal/professional domain	*My brother he’s very conservative and he says whatever works for you…. I haven’t had any stigma.*
Stigma Outcomes: Affected Populations, Organizations and Institutions	Secrecy as means to mitigate stigma	In personal/professional domain	*To be honest with you, I’m still in the closet about my consumption… I decided… I would come out of the closet to a certain extent. And I do so with great caution.* *When my in-laws found out I was sort of horrified that they would have this bad perception of me… my husband has never smoked or done any marijuana in his life. But he’s fine with* [*it*]^*†*^
		In medical domain	*I didn’t* [*explain why I eat MC] because—I didn’t want to hear ‘well that’s foolish’ or ‘you can’t plan that to work’ or—you now what I mean. Something negative.*^†^ *There’s no advantage to telling* [*my doctor*]*.… Judgements. Maybe my company, health insurance could be affected by it. You know, the less documentation I feel like I have on it, the better.*^†^ *Right now, I haven’t told anybody* [*in my medical clinic*].
	Imperviousness as response to stigma	Global	*My attitude is I don’t care what they think…. So if they want to think I’m just some pothead, I don’t give a* [*expletive*]. *I just don’t*. *Yeah* [*I felt stigma*], *but I don’t care because I’m going to get well*.

*MC* medical cannabis

^†^Indicates examples of presumed rather than directly experienced stigma

Nearly half of participants discussed stigmatization in the medical domain. Sub-themes included the seemingly “taboo” nature of medical cannabis in the clinical setting (“It’s all hush hush [in the doctor’s office]”) and unwillingness of healthcare providers to engage in in-depth medical cannabis discussions. For instance, one participant expressed frustration over a medical professional labeling them as a “substance abuser” without further exploration of their medical cannabis use; another experienced “giggling” at a medical conference when they publicly disclosed medical cannabis use. Social judgment and prejudice emerged as powerful drivers for medical cannabis stigma and, notably, no participant denied such stigma in the medical setting.

Most participants perceived stigma in the personal/professional domain. Those who did noted a mixed response among friends and family, with some in their social network supportive of and others standing in opposition to their use. Those disapproving were seen to define medical cannabis as a “gateway drug” and its use as a substance use disorder, again suggesting social judgment and prejudice as powerful drivers. Some participants indicated a “no-drug-use policy” from family members. Unlike in the medical arena, however, a quarter of participants—all from the Eastern United States—explicitly reported the absence of medical cannabis-related stigma in their personal and professional lives. In fact, a few noted that perceptions of medical cannabis were shifting and that the door to medical cannabis acceptance was “*creaking open*” suggesting that cultural norms were increasing less of a facilitator of stigma. Of note, stigma was perceived across the cancer trajectory including among those undergoing and those in the surveillance stage of cancer treatment.

### Some medical cannabis-related stigma internalized rather than directly experienced

While some manifestations of stigma were external, an emergent theme across both personal/professional and medical domains was that of internalized stigma. For instance, in the personal/professional domain, one participant noted:

*[My family] probably thinks … I’m a junkie.*

In the healthcare domain, another participant assumed their medical team’s response to medical cannabis use would be negative:

*I didn’t want to hear [from providers], ‘well that’s foolish or you can’t plan [medical cannabis] to work.’*

Yet, another reported:

*[Healthcare professionals] have a kind of moral opposition to [medical cannabis use]. I’m reading into it. I’m not quite sure if that’s really the case.*

### While many responded to medical cannabis-related stigma with secrecy, some were impervious

Common strategies among participants to mitigate stigma included sharing partial truths and being judicious about with whom to self-reveal. While all participants were open about medical cannabis use in the medical setting to some degree, most discussed medical cannabis with healthcare providers only generally, without disclosing full details (e.g., mode of consumption; types of medical cannabis products used). Many shared their medical cannabis use with a select few in their social circle, but deliberately refrained from informing those they feared might stigmatize their medical cannabis practice. A few participants reported being unphased by stigma and not allowing it to alter behavior. For example, one participant maintained:

*Yeah [I felt stigma], but I don’t care because I’m going to get well.*

These health/social impacts of stigma seemed ubiquitous in that judiciousness with one’s medical cannabis history was equally common among participants in active cancer treatment as those in the surveillance stage of cancer care.

## Discussion

In exploring whether individuals with cancer histories using medical cannabis experience stigma around this practice, we learned that medical cannabis-related stigma was perceived in medical and personal/professional domains but seemed more prevalent in the former. In fact, a quarter of those interviewed—all from the Eastern U.S.—denied any stigma in their personal lives. A sizeable proportion of the stigma discussed was presumed rather than directly experienced. While many participants responded to stigma with partial or complete secrecy, a few reported being impervious, altering neither transparency nor behavior, in other words, endorsing few health or social impacts. Taken together, these findings suggest that, as articulated by a study participant, the door to medical cannabis acceptance may be *creaking open* but seems to be doing so more gradually in the medical realm as compared to the personal/professional one.

### Clinical implications

Our findings extend those of earlier research investigations that pointed both to widespread medical cannabis-related stigma, as well as to possible early shifts in culture around medical cannabis. The fact that participants in our study frequently reported presumed stigma suggested that historic antecedents served as facilitators. The finding of regional differences in medical cannabis-related stigma is also of interest, particularly since this is, to our knowledge, the first study of medical cannabis-related stigma to capture a geographically diverse U.S. sample. One could hypothesize that local variations in legal/cultural/informational milieus might underlie these differences. Our study should be followed by a quantitative, longitudinal assessment of oncologic medical cannabis-related stigma in the U.S among patients, caregivers, and clinical care teams, in order to quantify regional differences, their etiologies, and trends over time. To fully capture the experiences of affected populations, a more comprehensive study should include individuals with cancer acquiring cannabis from medical cannabis dispensaries, adult use dispensaries as well as informal sources. If quantitative assessments support our findings, greater medical cannabis-related communication in the clinic may be necessary to improve transparency on the part of individuals with cancer histories regarding decisions about whether to use medical cannabis, modes of self-administration, ratios of active ingredients, target indications, potential risks, and adverse events, etc. One could imagine that an important component for improved communication would be strengthened cannabinoid therapeutics education for clinicians [10, 17].

### Study limitations

This study has important limitations. It is cross-sectional, a weakness given the rapid pace of change regarding state cannabis legalities. The sample, which consisted of individuals state-sanctioned to use medical cannabis, might differ in viewpoint from those accessing cannabis from adult use dispensaries or sans state approval. Our study specifically evaluated medical cannabis perceptions among individuals with cancer histories. Levels of perceived stigma may differ among individuals with other illnesses turning to medical cannabis.

## Conclusions

Our study also has important strengths. We achieved thematic saturation suggesting that our sample size did in fact allow for broader perceptions of medical cannabis-related stigma among cancer patients to be reflected. The study’s wide geographic sampling also strengthened its generalizability as compared to earlier studies on this topic. Finally, its key findings—that medical cannabis-related stigma, while highly prevalent, exists more in medical than in personal/professional domains and often leads to a degree of secrecy—will be of considerable interest to healthcare providers who depend on patient transparency when gathering medical history and in the devising optimal care plans.
